# Characterization of Defocused Coherent Imaging Systems with Periodic Objects

**DOI:** 10.3390/s24216885

**Published:** 2024-10-26

**Authors:** Gianlorenzo Massaro, Milena D’Angelo

**Affiliations:** 1 Dipartimento di Fisica, Università degli Studi di Bari, 70125 Bari, Italy; milena.dangelo@uniba.it; 2 Istituto Nazionale di Fisica Nucleare, Sezione di Bari, 70125 Bari, Italy

**Keywords:** coherent imaging, 3D imaging, optical resolution

## Abstract

Recent advancements in quantum and quantum-inspired imaging techniques have enabled high-resolution 3D imaging through photon correlations. These techniques exhibit reduced degradation of image resolution for out-of-focus samples compared to conventional methods (i.e., intensity-based incoherent imaging). A key advantage of these correlation-based approaches is their independence from the system numerical aperture (NA). Interestingly, both improved resolution of defocused images and NA-independent scaling are linked to the spatial coherence of light. This suggests that while correlation measurements exploit spatial coherence, they are not essential for achieving this imaging advantage. This discovery has led to the development of optical systems that achieve similar performance by using spatially coherent illumination and relying on intensity measurements: direct 3D imaging with NA-independent resolution was recently demonstrated in a correlation-free setup using LED light. Here, we explore the physics behind the enhanced performance of defocused coherent imaging, showing that it arises from the modification of the sample’s spatial harmonic content due to diffraction, unlike the blurring seen in conventional imaging. The results we present are crucial for understanding the implications of the physical differences between coherent and incoherent imaging, and are expected to pave the way for the practical application of the discovered phenomena.

## 1. Introduction

The development of new quantum and quantum-inspired unconventional imaging techniques has led to exploration of the possibility of performing high-resolution 3D imaging exploiting light intensity and photon correlations [1,2,3]. In particular, recent developments demonstrate that, when imaging a sample placed out of focus (i.e., in a plane different from the conjugate plane of the detector), the degradation of the resolution occurs on a much larger axial scale compared to conventional incoherent imaging. Remarkably, such correlation-based techniques exhibit the unprecedented feature of being independent of the numerical aperture (NA) of the optical design [4,5,6,7,8]. The physical origin of such peculiar optical performance has recently been found to be a consequence of the *spatial* coherence [9] of light involved in the process of image formation [6]. In fact, images recovered by such correlation-based methods can be exactly reduced to images collected by an imaging system illuminated by coherent light [10]. Correlations can thus be seen as one of the possible tools for accessing, and exploiting, the optical performance granted by spatial coherence. In other words, the same optical performance of the aforementioned quantum imaging techniques can be achieved through conventional intensity measurements, performed in well-thought optical systems designed to exploit the spatial coherence of the illumination source. However, due to the need to rely on the spatial coherence of the illumination light, this effect can only be exploited in the context of brightfield imaging. This has recently been demonstrated, both theoretically and experimentally, in the context of microscopy. In fact, direct 3D imaging with spatially coherent illumination has been achieved by employing light from an LED array, obtaining NA-independent lateral resolution in a correlation-free imaging system [11].

Conventional imaging applications typically favor illumination from a spatially incoherent source, as is the case with bright-field imaging [12]. Its ease of use, cost-effectiveness and fast acquisition are among the reasons of the widespread use of incoherent imaging. Being a direct technique, namely, an imaging method that does not rely on severe post-processing of the information acquired by the sensor, incoherent imaging is capable of real-time capturing and visualization. Devices based on incoherent illumination are extremely convenient when working with well-focused flat samples, i.e., having negligible thickness along the optical axis. However, in the presence of defocusing and thick samples, sensitivity to the planes outside of the depth of field (DOF) of the imaging apparatus is lost due to blurring [13,14]. Blurring of out-of-focus plane is notoriously caused by the NA-dependent circle of confusion (CoC), which results in a strong trade-off between the achievable resolution and DOF [15,16]. Such trade-off is even more critical when single-shot 3D imaging of thick samples is needed, namely, when performing light-field imaging [17,18,19]. In fact, the possibility of performing post-processing axial sectioning of the sample comes, in light-field imaging, at the expenses of a reduced resolution and an even stronger trade-off between resolution and DOF. Furthermore, the optical performance is always dictated by the NA of the optical design [20]. As recently demonstrated, illumination through spatially coherent light is a viable direct imaging method to achieve brightfield light-field imaging, overcoming the resolution-DOF compromise typical of conventional light-field, as performed with incoherent light [11].

The use of coherent illumination for direct imaging applications is much less frequent compared to the case of *inverse* techniques, which reconstruct the sample through inverse computation methods and algorithms, applied on the collected intensity [21,22,23]. Notable examples of inverse imaging techniques are holography [24,25,26] and ptychography [27,28,29,30]. These techniques, which are sometimes used in conjunction with direct methods, aim at collecting information that is typically not available with direct methods, and can thus achieve super-resolution [31,32], wavefront reconstruction [33], and correction of optical aberrations [23], at the expense of image reconstruction time [34]. The primary factor limiting the widespread use of coherent illumination in direct imaging is the noticeable fringing effects it introduces in the output images [35,36,37], that are typically regarded as artifacts. However, as recently demonstrated, the coherence-induced artifacts affect the images in a way that does not prevent coherent illumination from being used for direct imaging [11]. Its limited use is thus also partly due to the lack of a thorough understanding of the effects of coherence on the images, as well as to the inadequacy of the mathematical tools and methods used to assess the performance of coherent imaging systems. For these reasons, the use of coherent light has so far been confined to specific use cases: coherence is used to probe specific object features [38,39], rather than for general-purpose wide-field imaging, aimed at reproducing an object as faithfully as possible.

As we shall prove in this paper, coherent illumination can be used as a powerful tool to enhance the *direct* imaging resolution of an optical design, regardless of the application. We show that, for a given object size, the axial range in which an imaging system can retrieve a faithful image of the sample is much larger when illuminating with coherent light, compared to the conventionally used incoherent illumination (e.g., Kohler illumination). Furthermore, such DOF extension is independent of the NA. On the other hand, focused images obtained by spatially coherent illumination are still characterized by a NA-dependent Rayleigh-limited resolution, in perfect analogy with the case of incoherent illumination [40]. Hence, coherent illumination is shown to be a useful choice even when the richness of the information contained in the interference fringes is not exploited by complex reconstruction algorithms, as is done in indirect imaging.

This work strengthens the validity of the results of Ref. [11] and deepens our understanding of the physical reason behind the improved performance of coherent systems. In fact, a significant result is the development of a formal context to demonstrate that the optical performance of defocused coherent imaging can be brought back to the diffraction of the electric field from the sample plane to the plane in focus. This physical phenomenon is, as expected, completely independent on the particular imaging device and its numerical aperture. In fact, the presented analysis of imaging of periodic objects allows us to demonstrate that the NA causes losses in the amplitude of frequency components only below the Rayleigh limit at focus; however, such losses are generally negligible when considering defocused imaging. In addition, diffraction is found to alter the sample spatial frequency content by adding higher harmonics, making it the primary factor ultimately limiting the resolution of coherent imaging systems. The results we present are essential for understanding the physical differences between coherent and incoherent imaging, and are anticipated to guide the practical application of the phenomena we have discovered.

The paper is structured as follows. In Section 2, we lay the formal ground that allows us to carry out a comparison between the optical performance of coherent and incoherent systems; since our analysis focuses on periodic samples, we shall investigate a class of object whose electric field transmission function can be expanded into a Fourier series. At the end of the section, we show that the formalism we developed retrieves all the well-known properties of defocused incoherent imaging systems. The corresponding analysis, extended to the unknown case of a coherent system, is developed in Section 3, where we show the deep differences between the two imaging modalities. The results are discussed in Section 4, where the concept of modulation transfer functions (MTF) is appropriately extended to coherent imaging, allowing for a direct comparison with the performance of incoherent systems. In Section 5, we employ the formalism developed in the previous section to estimate estimate the imaging resolution of coherent systems, to confirm that an illumination strategy based on coherent light is an effective tool for improving the defocused performance of an imaging device.

## 2. Materials and Methods

### 2.1. Optical Response of Coherent and Incoherent Imaging Systems

**Figure 1:**
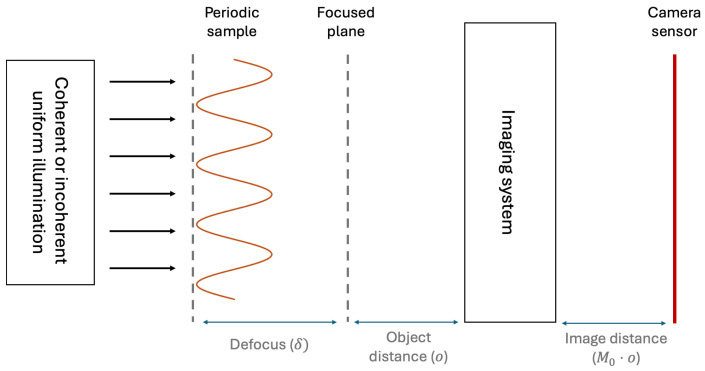
General scheme of the imaging device.

When an object is observed through an imaging device, the characteristics of the image captured by a photodetector vary significantly depending on whether the sample is illuminated with coherent or incoherent light [35]. The reason behind this can be found in the different way the field Green’s functions enter into the image formation process. In fact, the propagation of the electric field from the sample transverse plane, through the optical setup, and up to detector is described by the same Green’s function in both cases:

(1) $$P_{\delta }\left(x_{s},x\right)=\int D_{\delta }\left(x_{s}-x_{0}\right)\;P_{0}\left(x_{0},\frac{x}{M}\right)\;dx_{0},$$

where $x_{s}$ and *x* denote the transverse plane coordinates at the sample and detector planes, respectively; δ denotes the axial distance of the sample from focus; $P_{0}$ denotes the *complex* Green’s function of the focused imaging system, where $x_{0}$ is the transverse coordinate on the plane in focus, and *M* is the magnification of the imaging system at focus; $D_{\delta }$ is the vacuum Green’s function describing the electric field propagation from the sample plane to the plane in focus by an axial distance δ, supposed positive if the object is defocused away from the imaging system, and negative if defocused towards it (see Figure 1). Thus, field propagation in a defocused system can always by decomposed into the free-space propagation of the field towards the plane in focus, then convolved with the response function of the optical system. In this paper, we shall deal with paraxial imaging systems [35], so that the vacuum Green’s function has the convenient Gaussian form

(2) $$D_{\delta }\left(x_{s}-x_{0}\right)=\frac{e^{i\;k\delta }}{\sqrt{i\frac{2\pi \delta }{k}}}\;\exp\left[i\frac{k}{2\delta }{\left(x_{s}-x_{0}\right)}^{2}\right],$$

where k=2π/λ is the wavenumber of the illumination light, supposed monochromatic. As expected from a focused imaging system (i.e., with δ=0), when the sample is in focus, Equation (1) becomes

(3) $$P_{\delta =0}\left(x_{s},x\right)=P_{0}\left(x_{s},\frac{x}{M}\right).$$

Let us now see how the field propagator of Equation (1) enters into the image formation process, in the two cases of coherent and incoherent illumination. Assuming the object has a complex electric field transmittance A(x), the coherent image $I_{\mathrm{coh}}\left(x\right)$ obtained by shining spatially coherent plane-wave illumination onto the sample reads

(4) $$I_{\mathrm{coh}}\left(x\right)={\left|\int A\left(x_{s}\right)\;P_{\delta }\left(x_{s},x\right)\;dx_{s}\right|}^{2}=\int \int A\left(x_{s}\right)A^{*}\left(x_{s}^{\prime }\right)P_{\delta }\left(x_{s},x\right)P_{\delta }^{*}\left(x_{s}^{\prime },x\right)\;dx_{s}dx_{s}^{\prime },$$

whereas an incoherent image $I_{\mathrm{inc}}\left(x\right)$ of the same object reads

(5) $$I_{\mathrm{inc}}\left(x\right)=\int {\left|A(x_{s})\right|}^{2}{\left|P_{\delta }\left(x_{s},x\right)\right|}^{2}dx_{s}.$$

The formal differences between the two expressions are rather obvious: coherent imaging is a phase-sensitive technique, due to its responsiveness to the *complex* transmittance of the sample; incoherent imaging, conversely, is only sensitive to the *intensity* profile of the sample, i.e., the square module of the transmittance. In this work, we aim to establish common ground for understanding the intrinsic differences between coherent and incoherent imaging. To this end, we will examine the optical response of both imaging modalities to real-valued periodic patterns. This choice is customary in modulation transfer function (MTF)-like characterization [41], where imaging systems are characterized in terms of the extent in which they attenuate the spatial frequency content of a periodic object.

Another important consideration arising from the comparison between Equations (4) and (5), which has deep influence on the topic of this work, is that incoherent imaging is a *linear* image formation process: the incoherent image of the sample ${\left|A(x_{s})\right|}^{2}$ is given by a convolution of the sample with a real-valued positive point-spread function (PSF) ${\left|P_{\delta }\left(x_{s},x\right)\right|}^{2}$ [35]. In other words, at any given image coordinate *x*, the final image can be interpreted as a superposition of the intensity at the object plane ${\left|A(x_{s})\right|}^{2}$, weighed by the $x_{s}$-dependent coefficients $f_{\mathrm{inc}}\left(x_{s}\right)={\left|P_{\delta }\left(x_{s},x\right)\right|}^{2}$. Although trivial, such property of incoherent imaging does not have a counterpart in the case of coherent illumination. In fact, the coherent image formation process can be recognized a non-linear phenomenon, and *cannot* be described by a point-spread function. From Equation (4), we see that the image coordinate *x* is not given by a linear superposition of the intensity at the sample plane (convolution), but, rather, by the two-point interference between the electric field a points $x_{s}$ and $x_{s}^{\prime }$, weighed by a “second-order” *complex* response function $f_{\mathrm{coh}}\left(x_{s},x_{s}^{\prime }\right)=P_{\delta }\left(x_{s},x\right)P_{\delta }^{*}\left(x_{s}^{\prime },x\right)$. The most obvious consequence on the non-linearity of coherent imaging is that most image quality estimators conventionally used to assess the optical quality of incoherent images cannot be as effectively translated as quality estimators in the case of coherent imaging, as discussed in Refs. [6,11,42] and made evident in the rest of this paper.

### 2.2. Optical Response of Incoherent Systems to a Periodic Signal

The linearity of incoherent imaging systems enables the use of a wide range of simple image quality estimators to fully characterize the optical performance of a given imaging device; PSF, two-point resolution, knife-edge, and MTF are among the most widely used [43,44]. However, these imaging performance evaluation methods are all based on the fact that the image of a sample is blurred (or spread) by the optical setup. In fact, this kind of description of the behavior of a system is only applicable to *linear* systems, i.e., systems responding to a real-valued stimulus through a convolution with a real-valued PSF. However, the non-linearity of coherent imaging prevents the definition and use of such estimators, as both the PSF (from which the concept of two-point resolution is derived) and MTF lose their physical and mathematical meaning.

We shall recall a few mathematical aspects showing why the MTF is commonly used as an image quality estimator for standard imaging. To this end, let us consider a sample with a periodic intensity profile in the transverse plane, whose period is 1/ν. Its intensity profile can be expressed in terms of its Fourier series [45]

(6) $${\left|A_{\nu }\left(x_{s}\right)\right|}^{2}=\sum_{n=-N}^{N}a_{n}\;e^{i2\pi \;n\nu \;x_{s}},$$

where we have assumed 1/nν to be the highest harmonic content of the sample and $a_{n}$, with n=−N,⋯,+N, to be the coefficients of the Fourier expansion. From Equation (5), we see that the image of such object is given by

(7) $$I_{\mathrm{inc}}\left(x\right)=\sum_{n=-N}^{N}a_{n}\frac{\Phi_{\delta }\left(n\nu \right)}{2\pi }\;e^{i2\pi \;n\nu \;x/M(\delta )},$$

where M(δ) is the magnification, and

(8) $$\Phi_{\delta }\left(\nu \right)=\int {\left|P_{\delta }\left(x_{s}^{\prime }\right)\right|}^{2}e^{-2\pi i\nu x_{s}^{\prime }}dx_{s}^{\prime }$$

is the Fourier transform of the *incoherent* PSF of the system. As we shall see in Section 3, the implicit dependence of ${\left|P_{\delta }\left(x_{s},x\right)\right|}^{2}$ on its two variables is of the form ${\left|P_{\delta }\left(x_{s},x\right)\right|}^{2}={\left|P_{\delta }\left(x_{s}-\frac{x}{M(\delta )}\right)\right|}^{2}$. Hence, being a one-variable function, the definition of the Fourier transform of Equation (8) in unambiguous. The image of a periodic object thus has the same harmonic content as the input function; however, the spatial frequency coefficients are modified differently along the spectrum, as established by the expression of the filtering function $\Phi_{\delta }\left(\nu \right)$. In most cases of interest, the MTF $\left|\Phi_{\delta }\left(\nu \right)/2\pi \right|$ is a decreasing function of the spatial frequency ν, so that the PSF acts as a δ-dependent low-pass filter. In summary, the difference between the image $I_{\mathrm{inc}}\left(x\right)$ and the input function ${\left|A_{\nu }\left(x_{s}\right)\right|}^{2}$ is a loss of visibility (attenuation) of the periodic details, with the higher spatial frequencies typically *blurred* more severely than the lower-frequency modulations.

#### Example of Incoherent Imaging with Gaussian Apodization

In order to show some qualitative reference results, we shall now report the optical performance of an incoherent imaging system, characterized in terms of its MTF. We shall deal with the simple case of an imaging system with Gaussian apodization: we shall assume that the pupil of the limiting optical element is treated so as to produce a Gaussian PSF, in order to have analytical expressions for all the formulas. This assumption, however, does not entail any loss of generality in our discussion. The imaging system has been modeled as having $M_{0}$ magnification at focus, and native numerical aperture ${\mathrm{NA}}_{0}=\sigma_{l}/o$, where $\sigma_{l}$ is the width of the Gaussian-apodized limiting iris, and *o* is the object distance of the focused plane. Since our analysis will deal with *defocused* samples, such a distance must not be confused with the axial position of the sample: the sample is placed at a generic distance o+δ, and its position relative to the focused plane is δ (see Figure 1). The PSF of the system thus reads

(9) $${\left|P_{\delta }\left(x_{s},x\right)\right|}^{2}=\exp\left[-\frac{{\left(x_{s}-\frac{x}{M(\delta )}\right)}^{2}}{2\;\sigma_{\mathrm{inc}}^{2}\left(\delta \right)}\right],$$

where $M\left(\delta \right)=M_{0}/\left(1+\delta /o\right)$ is the magnification of the imaging system, deviating from the native magnification as a function of defocusing, and

(10) $$\sigma_{\mathrm{inc}}^{2}\left(\delta \right)={\left(\frac{1}{4\pi }\frac{\lambda }{\mathrm{NA}(\delta )}\right)}^{2}+{\left(\left|\delta \right|\;\mathrm{NA}\left(\delta \right)\right)}^{2}=\sigma_{0}^{2}\left(\delta \right)+\sigma_{\mathrm{CoC}}^{2}\left(\delta \right)$$

The first contribution to the width of the PSF $\sigma_{\mathrm{inc}}$ is the Airy disk, as determined by the *effective* numerical aperture $\mathrm{NA}(\delta )=\sigma_{l}/\left(o+\delta \right)$, whereas the second can be easily recognized as the circle of confusion (CoC), whose radius is knowingly proportional to both the defocusing and the NA of the system.

The limitation to the optical performance of standard systems is thus determined the diffraction limit (Airy disk) close to focus, albeit the factor solely responsible for image degradation in the presence of defocusing is the geometrical CoC. This is due to the fact that the term $\sigma_{0}$ is negligible compared to $\sigma_{\mathrm{CoC}}$ when $\left|\delta \right|\neq 0$.

From Equation (9), it directly follows that the normalized MTF of the imaging system acts as a low-pass frequency filter

(11) $$\left|\frac{\Phi_{\delta }\left(\nu \right)}{2\pi }\right|=\exp\left[-\frac{\sigma_{\mathrm{inc}}^{2}\left(\delta \right)\;\nu^{2}}{2}\right];$$

The cut-off frequency of the low-pass filter at $1/e^{2}$ attenuation is

(12) $$\nu_{\mathrm{inc}}\left(\delta \right)=2/\sigma_{\mathrm{inc}}\left(\delta \right)=\left\{\begin{array}{cc} 2k\;{\mathrm{NA}}_{0} & \mathrm{when}\;\delta ≃0\\ \frac{2}{\left|\delta \right|\;\mathrm{NA}} & \mathrm{when}\;\delta \neq 0 \end{array}\right.$$

In the next section, we shall show that by applying the same formalism to the case of coherent illuminations, an exact counterpart to the MTF cannot be found. However, a comparison with incoherent imaging is still possible by considering how the coefficients of the Fourier expansion of the coherent image are modified by defocusing. The results thus enable a deeper understanding of coherent imaging and reveal the physical reasons behind its peculiar behavior.

## 3. Results

### 3.1. Optical Response of Coherent Imaging Systems to a Periodic Signal

The behavior of defocused coherent imaging systems to a periodic input function is extremely different from the response of standard (incoherent) imaging. Let us consider a sample with the same periodic profile as defined in Equation (6). Since coherent imaging is sensitive to the *field* transmittance, we shall assume $a_{-N},\cdots ,a_{+N}$ to be the Fourier expansion coefficients of $A_{\nu }\left(x_{s}\right)$, namely, the sample *field* transmittance. By applying Equation (4) and the Green’s function of Equation (1), one obtains the analytical expression for a defocused coherent imaging system:

(13) $$I_{\mathrm{coh}}\left(x\right)={\left|\sum_{n=-N}^{+N}a_{n}\frac{\phi_{0}\left(n\nu \right)}{2\pi }e^{-i\frac{z^{2}\left(\delta \right)\;n^{2}\nu^{2}}{2}}\;e^{i2\pi \;n\nu \;x/M_{0}}\right|}^{2},$$

where $M_{0}$ is the native magnification of the imaging system at focus and

(14) $$\begin{array}{cc} \phi_{0}\left(\nu \right) & =\int P_{0}\left(x_{0},x/M\right)e^{-2\pi i\nu x_{0}}dx_{0} \end{array}$$

(15) $$\begin{array}{cc} z^{2}\left(\delta \right) & =2\pi \lambda \;\delta . \end{array}$$

In the case of coherent imaging, the argument of $P_{0}$ being a single-variable cannot be applied, as we did for $\left|P_{\delta }\right|$ in the case of an incoherent system. In fact, unlike its squared modulus, the Green’s function $P_{\delta }$ includes some quadratic phase terms that do not depend on the difference between the two coordinates, namely, $P_{\delta }\left(x_{s},x\right)=\left|P_{\delta }\left(x_{s}-x/M\right)\right|\exp i\left(\alpha x_{s}^{2}+\beta x^{2}/M^{2}\right)$. For this reason, a more formally correct way of expressing Equation (14) would be to carry an explicit dependence on the image coordinate $\phi_{0}\left(\nu \right)=\phi_{0}\left(\nu ,x_{s}\right)$, in agreement to the definition. As a consequence of the *x*-dependence of $\phi_{0}$, Equation (13) cannot be considered as a true Fourier expansion, since some of the spatial dependence of the image is not decomposed into its harmonic content. In fact, because of the wavefront limitation imposed by finite optical components, the final image is not periodic, so that it cannot be expressed in a Fourier series. However, the *x*-dependence of $\phi_{0}$ is weak and can often be neglected, as discussed in Section Coherent Imaging with a Gaussian-Apodized System.

Even before considering the non-linear behavior of Equation (13), several differences are immediately noticeable, compared to the incoherent case (Equations (7) and (8)). Firstly, since $z^{2}\left(\delta \right)$ is a real number, $\left|\phi_{0}\left(n\nu \right)\exp\left(-i\;z^{2}\left(\delta \right)\;n^{2}\nu^{2}/2\right)\right|=\left|\phi_{0}\left(n\nu \right)\right|$. Therefore, the attenuation of the coefficients of the harmonic series depends on the cut-off frequency of the imaging system *in focus* (Airy disk). This is a key difference compared to the frequency filtering operated by $\Phi_{\delta }$, which imposes diffraction-limited low-pass filtering only in focus. In coherent systems, low-pass filtering associated to the attenuation of harmonic content is completely independent on the CoC; in other words, any harmonic component that is below cut-off at focus does not experience blurring when defocused, regardless how severe the defocusing. Conversely, the effect of defocusing has deep consequences on the phase of the expansion coefficients. This is one of the main results of this work, and we shall carefully prove it by reporting the complete Fourier expansion of Equation (13). The expression is obtained by expanding the squared modulus, thus obtaining a new Fourier series

(16) $$I_{\mathrm{coh}}\left(x\right)=\sum_{n=-2N}^{+2N}a_{n}^{\prime }\left(\delta \right)\;e^{i2\pi \;n\nu \;x/M_{0}}$$

with

$$a_{n}^{\prime }\left(\delta \right)=e^{-i\frac{z^{2}\left(\delta \right)\;n^{2}\nu^{2}}{2}}\sum_{m=-N}^{+N}a_{m}\frac{\phi_{0}\left(m\nu \right)}{2\pi }a_{n-m}\frac{\phi_{0}\left((n-m)\nu \right)}{2\pi }e^{iz^{2}v^{2}nm},$$

where $a_{\left|m\right|>N}=0$. The equation can be immediately recognized as the Fourier expansion of a function containing a way richer harmonic content compared to the input function, which extends up to the second harmonic 2Nν of the highest frequency Nν available in the original object.

### 3.2. Coherent Imaging of a Sinusoidal Function

In order to draw some more practical conclusion, we shall now examine the case of a sample containing very modest harmonic content. In fact, Equation (16) has a rather cumbersome expression even in the case of very simple samples, as we shall see shortly. For this reason, the interesting features of coherent imaging can be examined by considering the easiest non-phase periodic sample, namely, a positive sinusoidal oscillation with spatial period 1/ν

(17) $$S\left(x_{s};\nu \right)=\sin^{2}\left(\pi \nu x_{s}\right)=\frac{1}{2}\left[1-\cos\left(2\pi \nu x_{s}\right)\right].$$

By applying Equation (16), we obtain the coherent image of $S_{\nu }$ as a function of the parameters δ and ν

(18) $$\begin{array}{cc} I_{\mathrm{coh}}\left(x;\;\delta ,\nu \right)=1 & +\frac{1}{2}\frac{{\left|\phi_{0}\left(\nu \right)\right|}^{2}}{{\left|\phi_{0}\left(0\right)\right|}^{2}}\\ & -2\frac{\left|\phi_{0}\left(\nu \right)\right|}{\left|\phi_{0}\left(0\right)\right|}\cos\left(\frac{z^{2}\left(\delta \right)\nu^{2}}{2}+\varphi_{0}\left(0\right)\right)\cos\left(2\pi \nu x+\varphi_{0}\left(\nu \right)\right)\\ & \;\;\;\;\;\;\;\;\;\;\;\;\;\;\;+\frac{1}{2}\frac{{\left|\phi_{0}\left(\nu \right)\right|}^{2}}{{\left|\phi_{0}\left(0\right)\right|}^{2}}\cos\left(4\pi \nu x+\varphi_{0}\left(\nu \right)\right), \end{array}$$

where $\varphi_{0}\left(\nu \right)=\arg\phi_{0}\left(\nu \right)$. In this simple case, studying the effect of the δ-dependent phase of the coefficient of the Fourier expansion in Equation (18) is straightforward. To neglect the effect of low-pass filtering operated by the PSF of the focused system, let us assume the fundamental frequency of the sample to be much lower than the $\left|\phi_{0}\left(\nu \right)\right|$ filter cut-off. In these conditions, the imaging system can be assumed to be ideal, namely, $\phi_{0}\left(\nu \right)=1\cdot e^{i\cdot 0}$. This is the response function obtained in the limit of an infinite numerical aperture of the imaging system. Therefore, provided infinite-aperture approximation can be applied at the fundamental frequency of the sample, the image retrieved by a coherent imaging system as a function of defocusing reads:

(19) $$I_{\mathrm{coh}}^{\infty }\left(x;\delta ,\nu \right)=\frac{3}{2}-2\cos\left(\pi \lambda \delta \;\nu^{2}\right)\cos\left(2\pi \nu \;x\right)+\frac{1}{2}\cos\left(4\pi \nu \;x\right).$$

We should specify that this formula is an excellent approximation whenever the fundamental frequency is much larger than the cut-off frequency defined by the Airy disk, and is unaffected by the CoC. In these conditions the analysis of the features of the image obtained by a coherent system is straightforward:

- When the sample is in focus, or whenever δ is an even multiple of the quantity $1/\lambda \nu^{2}$,
  $$I_{\mathrm{coh}}^{\infty }\left(x;\;\delta =m\frac{2}{\lambda \nu^{2}},\nu \right)=\sin^{4}\left(\pi \nu \;x\right)=S^{2}\left(x_{s};\nu \right),$$
  with m∈Z. This situation thus corresponds to the condition of perfect imaging, in which the imaging system correctly reproduces the absolute squared image of the input sample.
- When the defocusing is an odd multiple of $1/\lambda \nu^{2}$,
  $$I_{\mathrm{coh}}^{\infty }\left(x;\;\delta =\frac{2m+1}{\lambda \nu^{2}},\nu \right)=\cos^{4}\left(\pi \nu \;x\right)=S^{2}\left(x_{s}+\frac{1}{2\nu };\nu \right).$$
  The image preserves the harmonic content of the original signal, but is shifted by half a period compared to the correct imaging condition.
- When $\delta =\left(m+1/2\right)/\lambda \nu^{2}$,
  $$I_{\mathrm{coh}}^{\infty }\left(x;\;\delta =\frac{m+1/2}{\lambda \nu^{2}},\nu \right)=\frac{3}{2}+\frac{1}{2}\cos\left(4\pi \nu \;x\right),$$
  modulation at the fundamental frequency ν of the original signal even disappears from the harmonic content of the image.

Another interesting feature of Equation (19) is that the period of the modulation of the harmonic content of the sample as a function of δ has a very strong dependence on the fundamental frequency ν, being inversely proportional to $\nu^{2}$, and not influenced by the NA of the system. This aspect is in strong contrast with the behavior of the incoherent imaging case, where the cut-off frequency attenuating the high-frequency content of the image is independent of ν and entirely defined by NA (see Equation (11)). As we shall see in the next section, in the specific case of Gaussian apodization, such conclusions are not a consequence of the infinite-NA approximation used to obtain Equation (19); on the contrary, with the due considerations, they are valid also in the context of non-ideal imaging.

#### Coherent Imaging with a Gaussian-Apodized System

The complex Green’s function of the Gaussian-apodized focused coherent system, already introduced in Section Example of Incoherent Imaging with Gaussian Apodization, reads

(20) $$P_{0}\left(x_{s},x\right)=\exp\left[-\frac{{\left(x_{s}-x/M_{0}\right)}^{2}}{4\sigma_{0}^{2}}\right]\exp\left[\frac{\pi i}{\lambda o}\left(x_{s}^{2}-\frac{x^{2}}{M_{0}^{2}}\right)\right],$$

where $\sigma_{0}$ is the Airy disk in the focused plane (as in Equation (10)). All the parameters, such as the object distance *o* and the lens width $\sigma_{l}$ are chosen to match the choices of Section Example of Incoherent Imaging with Gaussian Apodization. It should be noticed that, in accordance with the analysis developed in Section Example of Incoherent Imaging with Gaussian Apodization, the squared module of Equation (20) corresponds to the PSF of the focused incoherent imaging system (Equation (9)).

By applying Equations (14) and (16) to the sinusoidal sample $S_{\nu }$, the complete expression of its coherent image is obtained, reading

(21) $$\begin{array}{c} I_{\mathrm{coh}}\left(x;\;\delta ,\nu \right)=\exp\left[-\frac{x^{2}/M_{0}^{2}}{2\left(\sigma_{0}^{2}+\sigma_{l}^{2}\right)}\right]\left\{1\right.+\frac{c_{0}^{2}\left(\nu \right)}{2}\cosh\left(4\pi \nu^{\prime \prime }x\right)+\frac{c_{0}^{2}\left(\nu \right)}{2}\cos\left(4\pi \nu^{\prime }x\right)+\\ -2c_{1}\left(\nu \right)\cos\left[\pi \lambda \nu^{2}\left(\delta +\delta_{0}\right)\right]\cosh\left(2\pi \nu^{\prime \prime }x\right)\cos\left(2\pi \nu^{\prime }x\right)\\ \left.+2c_{1}\left(\nu \right)\sin\left[\pi \lambda \nu^{2}\left(\delta +\delta_{0}\right)\right]\sinh\left(2\pi \nu^{\prime \prime }x\right)\sin\left(2\pi \nu^{\prime }x\right)\right\}, \end{array}$$

where

$$\begin{array}{cc} c_{0}\left(\nu \right) & =\exp\left[-\frac{4\pi^{2}\nu^{2}}{\sigma_{l}^{-2}+\sigma_{o}^{-2}}\right]\\ \nu^{\prime } & =\frac{\nu }{1+\sigma_{0}^{2}/\sigma_{l}^{2}}\\ \nu^{\prime \prime } & =\frac{\nu }{\frac{o}{2k}\left(\sigma_{l}^{-2}+\sigma_{o}^{-2}\right)}\\ \delta_{0} & =\frac{o}{1+\sigma_{l}^{2}/\sigma_{0}^{2}}. \end{array}$$

The reparametrization has been chosen to carefully separate the ν-dependent terms that do not depend on δ, which are solely responsible for frequency filtering, from the *x*-dependent terms, which are responsible for space modulation, and the harmonic oscillations. Firstly, it should be noticed that, as expected from a plane-wave illuminated system with finite imaging apertures, the final image is no longer periodic, but has a limited field of view (FoV), as defined by the lens: as can be seen from the first factor in Equation (21), the coherent image of the periodic object has a Gaussian envelope, whose width is defined by the lens radius (in imaging, one typically has $\sigma_{l}\gg \sigma_{0}$). However, additional spatial modulation (representing spurious harmonic content) is introduced by the two hyperbolic sine and cosine functions which multiply the fundamental-frequency terms in the Fourier expansion, namely, $\cos\left(2\pi \nu^{\prime }x\right)$ and $\sin\left(2\pi \nu^{\prime }x\right)$. The spatial modulation of such functions occurs on the spatial scale $1/\nu^{\prime \prime }$; in typical imaging configurations, since both $\sigma_{l}\gg \sigma_{0}$ and $o\gg \sigma_{0}$, one has that $1/\nu^{\prime \prime }$ is much larger than the period of the sample. Hence, if we limit our analysis to a portion of the FoV that is reasonably far from the edges, both the Gaussian envelope and the cosh contributions can be neglected, whereas the sinh function is zero. In the middle of the FoV, the periodic nature of the coherent image is thus evident, and Equation (21) can formally be regarded as proper Fourier expansion. Compared to the ideal case, however, the fundamental frequency of the image is slightly modified by the imaging system, being reduced by a coefficient $1+\sigma_{0}^{2}/\sigma_{l}^{2}$. Once again, with conventional imaging parameters ($\sigma_{l}\gg \sigma_{0}$), such discrepancy can hardly be appreciated, so that $\nu^{\prime }≃\nu$, and, as such, also $\delta_{0}≃0$, while the cut-off frequency of the filtering function is entirely defined by the Airy disk. In imaging conditions and in the center of the FoV, the coherent image of a sinusoidal object thus reads

(22) $$\begin{array}{cc} I_{\mathrm{coh}}\left(x;\;\delta ,\nu \right)≃ & 1+\frac{1}{2}e^{-8\pi^{2}\sigma_{o}^{2}\nu^{2}}\left(1+\cos\left(4\pi \nu \;x/M_{0}\right)\right)+\\ \; & -2e^{-4\pi^{2}\sigma_{o}^{2}\nu^{2}}\cos\left[\pi \lambda \nu^{2}\delta \right]\cos\left(2\pi \nu \;x/M_{0}\right). \end{array}$$

**Figure 2:**
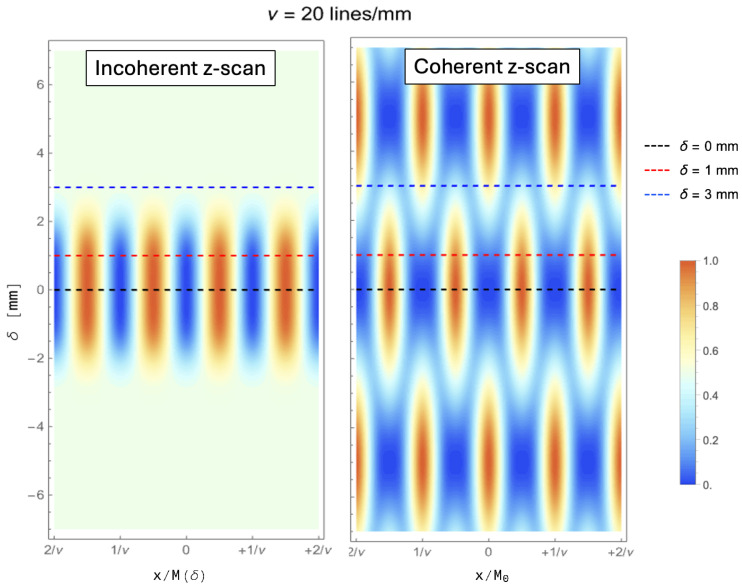
Comparison between the incoherent images (**left**) and coherent images (**right**) for a range of defocusing δ (vertical axis) from −7 mm to +7 mm. The plots are obtained by assuming a spatial frequency of ν=20 lines/mm, illumination at λ=500 nm and NA =0.05.

In Figure 2, we report a comparison between the incoherent images (left) and coherent images (right) that are obtained for a range of defocusing δ from −7 mm to +7 mm. The plots are obtained by assuming green illumination (λ=500 nm) and a Gaussian-equivalent NA $=\sigma_{l}/o$ of 0.05, resulting in a diffraction-limited cut-off frequency (see Equation (11)) of about $10^{5}$ cycles/mm. The plots for coherent imaging are obtained straight from Equation (21), without performing any approximations.

It is easily recognized that, since ν is much larger than the cutoff frequency, the behavior of the coherent case is extremely well predicted by Equation (19). In fact, coherent imaging shows periodic oscillations not only on the *x*-axis, as expected when imaging a sinusoidal object, but also on the δ-axis. Such oscillations are characteristic of the coherent case, as can be seen by direct comparison with the incoherent case. We shall now analyze the features of coherent imaging in further detail; we shall show that the behavior of such systems is easily explained by observing how the coefficient of the Fourier expansion evolves, as a function of defocusing.

## 4. Discussion

The properties of a conventional imaging device are extremely different when spatially coherent light is used to illuminate a periodic sample. The profound differences entailed by the two image formation processes are already evident by considering a sample with limited harmonic content, such as a positive sinusoidal oscillation with spatial period 1/ν. To compare the different performances of coherent and incoherent system in the case of this simple object, we report the analytical expression of the incoherent image when the intensity profile of the sample is ${\left|A(x_{s})\right|}^{2}=\sin\pi \nu x_{s}$:

(23) $$I_{\mathrm{inc}}\left(x;\;\delta ,\nu \right)=\frac{1}{2}\left[1-e^{-2\pi^{2}\left(\sigma_{0}^{2}\left(\delta \right)+\sigma_{\mathrm{CoC}}^{2}\left(\delta \right)\right)\nu^{2}}\cos\left(4\pi \nu \;x/M(\delta )\right)\right].$$

**Figure 3:**
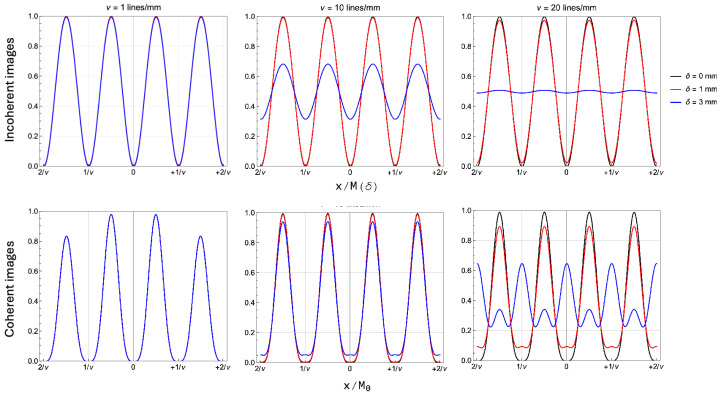
Comparison between the incoherent images ((**upper**) panels) and coherent images ((**lower**) panels) for three different spatial frequencies ν and three different defocusing values δ. The three spatial frequencies are ν=1 cycles/mm (**left**), 10 cycles/mm (**middle**), and 20 cycles/mm (**right**). In each panel, three different sample displacements are represented, corresponding to focus (black), +1 mm (red), and +3 mm (blue).

This expression has been used to evaluate the plot for the incoherent images reported in Figure 2. Figure 3 reports the different images obtained from the two types of illumination, for three values of the axial defocusing δ, and three values of the fundamental frequency ν. As for Figure 2, the plots are obtained by assuming green illumination (λ=500 nm) and a Gaussian-equivalent NA $=\sigma_{l}/o$ of 0.05. To better highlight the differences in defocused performance of the two systems, we have chosen to report the images generated by three spatial frequencies well above the Rayleigh limit, so as to discuss the features related to the lack of CoC in coherent images. As can be noticed from the largest spatial modulation we analyzed, namely ν=1 cycle/mm, both imaging methods are able of reproducing a perfect image on an extended region along the optical axis, resulting in a long depth of field (DoF). However, already at ν=10 cycles/mm, the difference in performance between the two techniques is evident, with the coherent image available with much better contrast at the largest defocus (δ=3 mm). However, the real difference in the nature of the imaging modalities itself is rather evident for the highest frequency we considered (right panels, ν=20 cycles/mm). The plots report the images at the three different values of δ reported in Figure 2, which can be used as a reference to understand the behavior of coherent imaging (lower panel). In fact, whereas the two techniques are both capable of perfect imaging in focus, already at the intermediate displacement of δ=1 mm, the image quality of coherent imaging seems to be lower than the incoherent analogous, in clear contrast with the expectation set by the middle panel. In fact, the physical origin of the image degradation process in the two cases is completely different, as already discussed above: the blurring and loss of contrast typical of standard imaging is entirely governed by the NA, but such effects do not exist with coherent illumination. Coherent image degradation with defocusing does not entail loss of contrast, as is evident by comparing the blue lines in the upper and lower panels, corresponding to the largest defocusing. However, the typically overlooked aspect of this peculiar behavior of coherent imaging is its strong dependence on the particular spatial frequency, which we shall now analyze.

Some of the features discussed above can be explained in a MTF-*like* context, extended to coherent imaging. However, as we discussed earlier, an MTF analysis is incorrect from a strictly formal point of view, and our discussion will also demonstrate that it is also a rather elaborate way of describing the performance of coherent imaging. In fact, due to the non-linearity of coherent imaging, a proper MTF cannot be defined: in the case of a linear system, the MTF is a *positive-valued* function (see Equations (7) and (11)), that depends both on the frequency and the defocusing. From Equation (16), we see that this is not the case for coherent imaging, where the coefficient of the expansion are modified both in amplitude (attenuation) and in phase. As we discussed, while the attenuation is independent of the defocusing, the phase-shift of the coefficients of the expansion critically depends on both the frequency and the defocusing; this is particularly evident if the Fourier series of a sinusoidal object is expressed in the cosine basis, as we do in Equation (18). From this equation, we see that a single function is not sufficient for describing how the coefficients of the expansion are modified by defocusing, so that an MTF-like analysis for the coherent case consists of analyzing the coefficients of the harmonic series independently. However, as will become evident in the following discussion, the coefficients have very different mathematical properties compared to the proper MTF of an incoherent system. To this end, one can consider the three functions

(24) $$\begin{array}{cc} {\mathrm{MTF}}_{\mathrm{inc}}\left(\delta ,\nu \right) & =e^{-2\pi^{2}\left(\sigma_{0}^{2}\left(\delta \right)+\sigma_{\mathrm{CoC}}^{2}\left(\delta \right)\right)\nu^{2}}\\ {\mathrm{MTF}}_{\mathrm{coh}}^{\left(1\right)}\left(\delta ,\nu \right) & =-e^{-4\pi^{2}\sigma_{o}^{2}\nu^{2}}\cos\left[\pi \lambda \nu^{2}\delta \right]=-{\mathrm{MTF}}_{\mathrm{inc}}^{2}\left(0,\nu \right)\cos\left[\pi \lambda \nu^{2}\delta \right]\\ {\mathrm{MTF}}_{\mathrm{coh}}^{\left(2\right)}\left(\delta ,\nu \right) & =e^{-8\pi^{2}\sigma_{o}^{2}\nu^{2}}={\mathrm{MTF}}_{\mathrm{inc}}^{4}\left(0,\nu \right), \end{array}$$

which can be easily recognized, from top to bottom, as the MTF of the incoherent system (Equation (23)), the coefficient of the fundamental frequency in the coherent Fourier expansion (Equation (22)), and the coefficient of the second harmonic. In our analysis, the last two functions are referred to as MTF, but they should be regarded as mere proxies for a *coherent* MTF. In fact, unlike a proper MTF, these functions can assume negative values. This property is compatible with the fact that the analysis of the coefficients does not represent a strict MTF analysis, whose goal is to study how the harmonic content is attenuated. Our analysis rather aims at understanding how the harmonic content of the image is modified and recombines, as a function of defocusing. From the rightmost equalities, we see that the main differences between the coherent and incoherent case are the absence of a δ-dependent CoC, acting as a *filter*, and the presence of an oscillating term, possibly negative-valued, for the fundamental frequency.

**Figure 4:**
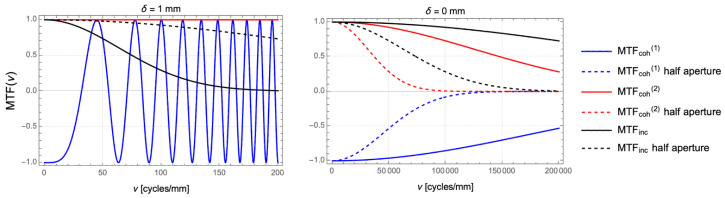
Comparison between the coherent and incoherent MTF as a function of the spatial frequency of the sinusoidal input for a 1-mm defocusing ((**left**) panel) and no defocusing ((**right**) panel). The solid lines represent the MTF, as obtained with the same NA as Figure 3, while the dashed lines are obtained with an halved NA of 0.025.

Figure 4 shows the trends of the MTF defined in Equations (24) at fixed defocusing, as a function of the spatial frequency of the sample. The left panel shows the case δ=1 mm, so as to compare with the qualitative results shown in Figure 3 for the same defocusing. By analyzing the solid lines, corresponding to the MTF at NA =0.05, one immediately recognizes that, in the range of frequencies explored in Figure 3, the three curves exhibit a different behavior: in the range [0,10] cycles/mm, both coherent MTF do not show significant deviation from their values in ν=0; on the other hand, by the upper end of the same frequency range, the incoherent MTF shows non-negligible frequency attenuation. The slower decay of the two coherent MTF in this range thus justifies the qualitative impression that coherent imaging is characterized by longer DOF compared to standard imaging. Starting from around ν=10 cycles/mm, however, the chirp of the fundamental MTF becomes non-negligible, with an oscillation period that increases linearly with the frequency. Such oscillations are at the origin of the seemingly unpredictable behavior of Figure 3 at $\nu =10^{2}$ cycles/mm, where the harmonic content of the coherent image is altered more severely than in incoherent imaging. In the right panel, we report evidence of the negligible effect of the NA in determining the optical features of coherent images. The dashed lines show the MTF obtained by halving the NA down to 0.025; the dashed lines corresponding to both coherent MTF are completely superimposed to the solid lines, so that they are not visible in the plot; on the contrary, a reduction of the NA improves the performance of a defocused incoherent system, as expected by a less severe CoC-induced blurring. The negligible dependence of the coherent MTF in the range of frequency we considered is easily explained by bearing in mind that the only NA dependence in the equation is contained in the term ${\mathrm{MTF}}_{\mathrm{inc}}^{2}\left(0,\nu \right)$, whose cut-off frequency is several orders of magnitude larger than the frequency range considered in the plots. This envelope thus becomes relevant only when the oscillating behavior of the MTF is not evident, namely, when $\cos\left[\pi \lambda \nu^{2}\delta \right]≃1$ for a long range of spatial frequencies. This can only happen around the plane in focus, provided $\nu^{2}\delta ≃0$. In this case, as reported in the left panel of Figure 4 for δ=0, the image properties of coherent imaging are analogous to those of conventional imaging, as described by the two NA-defined Airy disks.

The MTF-like analysis we have carried over has allowed us to obtain some quantitative results and give an explanation to many aspects of the peculiar features of images obtained through coherent illumination. However, the fundamental query of what is the maximum defocusing that is allowed to still retrieve a “good” image is easily answered only in the case of incoherent imaging: since the imaging system (either focused or defocused) acts as a δ-dependent frequency filter, the relationship between the frequency and the maximum defocusing it can undergo before being too blurred is obtained by requiring that, at any given δ,

(25) $$\nu \leq \nu_{\mathrm{inc}}\left(\delta \right),$$

where the cut-off frequency is expressed in Equation (11). The same argument cannot be applied to the coherent case, due to the lack of a monotonically decreasing filtering function. Furthermore, as we saw from Equation (19) and Figure 2, even the identification of a plane of best imaging is not straightforward for coherent imaging, due to the periodic occurrence of the same image as defocusing is changed. However, with the simple requirement that the plane of best imaging must be the same independently of the spatial frequency, only one plane is possible, namely, the plane in focus δ=0. The *frequency-dependent* DOF of coherent imaging should thus be defined as the largest axial range, in which the image of a sinusoidal object does change too much compared to the plane of best imaging. This requirement is analogous to the incoherent case, but, as we showed in great detail, the physical reason behind the loss of image fidelity for coherent imaging is not blurring, but the rearranging of the harmonic content in the image, as caused by defocusing. Being interested in assessing the *defocused* performance of coherent imaging, we can neglect the effect of the Airy disk, which, as we discussed, is only relevant in focus. Hence, changes to the harmonic content in the image of a sinusoidal object are only due to the factor $\cos\left[\pi \lambda \nu^{2}\delta \right]$, equal to 1 in the case of focused imaging (Equation (22)). Thus, by fixing a maximum value for the acceptable harmonic distortion to c⪆0, the parameter range for “good” imaging can be expressed as

(26) $$\left|\pi \lambda \nu^{2}\delta \right|\leq \arccos\left(1-c\right)≃\sqrt{2c}.$$

Up to irrelevant constants deriving from the arbitrary choice of what is considered to be a “good” image, we thus find that the relationship between a spatial frequency ν and the defocusing it can tolerate is given by

(27) $$\nu_{\mathrm{coh}}\leq \sqrt{\frac{1}{\lambda \;\left|\delta \right|}}.$$

This relationship confirms a recently proven fact, although with a completely different argument and test sample, namely, that the resolution of a coherent imaging system scales with a NA-independent square-root law of the absolute value of the defocusing [11]. To appreciate the improved defocused performance of coherent imaging, such a result must be compared with the analogous relationship for incoherent imaging; the analogous limit for incoherent illumination is obtained by imposing a tolerance to the maximum attenuation operated by the frequency filter, namely, $\exp\left(-\nu^{2}/2\nu_{inc}^{2}\left(\delta \right)\right)\geq 1-c$ (see Equation (11)). When δ≠0, this results in

(28) $$\nu_{\mathrm{inc}}\leq \frac{1}{\mathrm{NA}\left|\delta \right|}.$$

Hence, unlike the coherent case, the optical performance of incoherent imaging degrades with a well-known dependence on the NA. Furthermore, its performance has a less favorable tolerance to defocusing, being defined by an inverse proportionality law with defocusing, rather than inverse proportionality to its square root.

**Figure 5:**
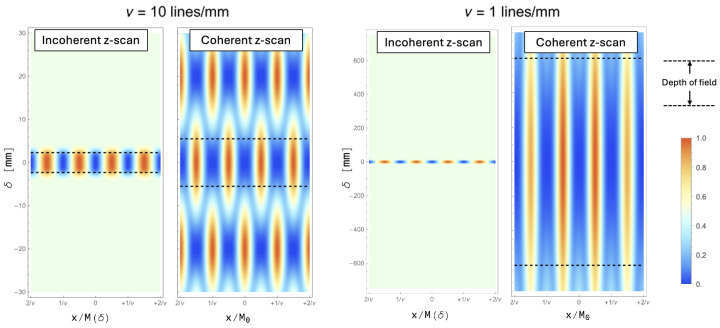
Comparison between the DOF of incoherent and coherent imaging for ν=1 lines/mm ((**right**) panel) and 10 lines/mm ((**left**) panel). The dashed lines identifying the DOF are obtained for a tolerance c=20%.

Figure 5 reports the DOF of coherent and incoherent imaging for the spatial frequencies ν=1 and 10 lines/mm. The DOF is evaluated by assuming c=20% tolerance on both the MTF of incoherent imaging, and on Equation (26) for coherent imaging. As can be seen also from comparison with Figure 2, the square-root scaling of coherent imaging makes it increasingly favorable over the incoherent case as larger spatial frequencies are considered.

## 5. Conclusions

The analysis carried out in this work has ultimately demonstrated that diffraction from the object plane to the plane in focus imposes a square-root scaling on the resolution of coherent imaging systems (Equation (27)). This result deserve particular emphasis, since it has been obtained through entirely different arguments, methodology, and class of objects compared to the original context in which it was first introduced and experimentally demonstrated [11]. In fact, it indicates that, regardless of the particular class of samples and image quality assessment procedure, spatially coherent illumination is a viable strategy to greatly improve the resolution of a defocused imaging system, and further emphasizes the remarkable property of being independent of the numerical aperture of the device.

On the downside, unlike traditional *direct* imaging techniques, which are mostly based on incoherent illumination or self-emitting samples, coherent illumination is characterized by extremely poor axial localization of the sample. This is a consequence of its entire harmonic content persisting along the optical axis without ever losing contrast, even repeating itself periodically (see Figure 2 and Figure 5). This feature makes the use of coherent illumination unpractical for application where a high-resolution image of the sample should also be integrated with information about the axial localization, as is the case for 3D imaging. However, a solution to this issue is already known: by exploiting illumination from multiple illumination points, tomographic information can be extracted in post-processing, thus blending the high-resolution advantage of coherent light and the fine axial localization enabled by high-NA tomographic systems [11]. The optical performance of such devices is, in fact, extremely similar to the 3D capabilities of quantum quantum-inspired correlation-based imaging modalities [6,42,46]. Translating this optical behavior outside of its original context of correlation-based imaging opens up to both a great simplification of the optical design, as well as to the possibility of performing imaging at a much higher signal-to-noise ratio [47,48,49,50,51,52,53]; both these aspects significantly contribute to make the technique compatible with real-time imaging of dynamical processes, and effectively competitive with state-of-the-art imaging modalities and sensors. Furthermore, conventional imaging scenarios impose much less demanding technological requirements to imaging sensors. In fact, quantum imaging typically involves correlation-based approaches imposing significant constraints on both the temporal performance of the detectors and their efficiency; the applicability of such techniques is thus strictly dependent on the available technology [54,55,56,57,58,59,60,61,62,63]. On the contrary, when exploiting the spatial coherence of light emitted by generic sources, such as LEDs or lasers, the optical performance improvement is achieved with standard detector technology, such as CMOS or CCD, with no need for dedicated sensors; specific requirements in the source, sensor, or setup, are thus only dictated by the application of interest, rather than by the imaging modality. On the downside, since the features of coherent imaging we described are inherently based upon detection of the coherent *illuminating* light, the advantages we discussed can be obtained only in the context of absorption imaging. Therefore, in order to achieve the same optical performance in contexts where the object is the emitter of photons or if brightfield imaging is not a viable option, the correlation-based architecture must be utilized. 

## Data Availability

Data are contained within the article.

## Abbreviations

The following abbreviations are used in this manuscript:

NA	Numerical aperture
DOF	Depth of field
CoC	Circle of confusion
MTF	Modulation transfer function
PSF	Point-spread function
